# Regulation of mTORC1 activity by the Golgi apparatus

**DOI:** 10.12703/r/10-50

**Published:** 2021-05-28

**Authors:** Christian Makhoul, Paul A Gleeson

**Affiliations:** 1 The Department of Biochemistry and Pharmacology and Bio21 Molecular Science and Biotechnology Institute, The University of Melbourne, Victoria 3010, Australia

**Keywords:** mTORC1, Golgi architecture, *trans*-Golgi network, actin, Rab1A, GOLPH3, GCC88, Arf1, GAT4, signalling

## Abstract

Mechanistic (or mammalian) target of rapamycin complex 1 (mTORC1) is a major signalling kinase in cells that regulates proliferation and metabolism and is controlled by extrinsic and intrinsic signals. The lysosome has received considerable attention as a major hub of mTORC1 activation. However, mTOR has also been located to a variety of other intracellular sites, indicating the possibility of spatial regulation of mTORC1 signalling within cells. In particular, there have been numerous recent reports of mTORC1 activation associated with the Golgi apparatus. Here, we review the evidence for the regulation of mTORC1 signalling at the Golgi in mammalian cells. mTORC1 signalling is closely linked to the morphology of the Golgi architecture; a number of Golgi membrane tethers/scaffolds that influence Golgi architecture in mammalian cells that directly or indirectly regulate mTORC1 activation have been identified. Perturbation of the Golgi mTORC1 pathway arising from fragmentation of the Golgi has been shown to promote oncogenesis. Here, we highlight the potential mechanisms for the activation mTORC1 at the Golgi, which is emerging as a major site for mTORC1 signalling.

## Background

A major signalling pathway in eukaryotic cells is the mechanistic (or mammalian) target of rapamycin (mTOR) pathway. mTOR is a Ser/Thr kinase that exists in two distinct complexes: mTORC1 and mTORC2. mTORC1 controls cell growth and metabolism, whereas mTORC2 regulates cytoskeletal dynamics^1^. mTORC1 senses the nutritional and energy status of a cell and initiates a variety of downstream responses by the phosphorylation of target substrates. Activation of mTORC1 promotes ribosome biogenesis, protein translation and nutrient import and also inhibits autophagy and stress-responsive transcription^2^. For example, active mTORC1 phosphorylates the downstream targets ribosomal S6 kinase and ribosome-associated eukaryotic translation initiation factor 4E-binding protein (4E-BP1) to drive protein translation^3^.

A number of inputs have been implicated in mTORC1 signalling, namely growth factors, nutrients, oxygen levels, energy levels and stress^4^. Surface receptors, such as insulin receptor, mediate activation of mTORC1 via phosphoinositide-3 kinase (PI3K)^4^. Nutrients, especially amino acids such as leucine, arginine and glutamine, activate mTORC1^5^. In addition, mTORC1 senses the energy status of a cell and is inhibited under conditions of energy deprivation via AMP-dependent kinase (AMPK)^6^. mTORC1 is also inhibited by hypoxia and other signals, including DNA damage^2,4^. Collectively, inputs regulate the activation and inhibition of mTORC1 and a variety of downstream targets regulate cellular processes. Understanding the regulation of this complex network of pathways is a key issue in cell biology.

## Hubs for mTOR

The lysosome is a well-known site/hub for amino acid sensing and activation of mTORC1^7^. The mechanism for lysosomal amino acid sensing involves the heterodimeric, small G protein, Rag GTPase, which binds to the surface of lysosomes via the pentameric Ragulator complex^8^. Amino acid availability leads to GTP loading of RagA or B which heterodimerises with GDP-bound RagC or D to form an active Rag complex which recruits mTORC1 to the lysosomal membrane surface and is activated in a Rheb GTPase–dependent manner^7^.

A remaining question in the field is whether the lysosomal hub controls the full repertoire of mTOR signalling outputs. It remains unknown whether the lysosomal hub controls all the downstream signalling responses. Indeed, evidence has emerged that mTOR is localised at a number of different intracellular sites in addition to lysosomes, such as the plasma membrane, secretory pathway, endosomes, mitochondria, peroxisomes and the nucleus^9,10^. The extensive distribution of mTOR on various organelles suggests that mTORC1 signalling may be regulated at multiple intracellular locations in a spatially specific manner. On a theoretical level, a multi-hub model would provide a greater capacity to fine-tune the complex network of mTOR signalling pathways, as is the case for other signalling pathways such as Toll-like receptor 4 (TLR4) activation^11^, where the cell surface and endosomes mediate different downstream outputs from the same receptor. Indeed, recent reports have provided evidence for spatially and functionally distinct pools of TORC1 at the vacuoles and endosomes in yeast^12^ and for activation of mTORC1 at the cell surface of mammalian cells independent of lysosomes^13^. Notably, there is also growing evidence for a role of the secretory pathway in the regulation of mTORC1 signalling, in particular the Golgi apparatus.

## Golgi as a general signalling hub

Many components of mTORC1 were initially identified by genetic screens in yeast^1^, an organism that lacks the detailed architecture of the vertebrate Golgi apparatus. In vertebrates, individual stacks of Golgi cisternae are fused together into a compact ribbon structure located in close proximity to the microtubule-organising centre^14^. The vertebrate Golgi was recently recognised to be associated with a range of higher-order cell functions in addition to the classic functions of glycosylation and membrane trafficking. The Golgi architecture is highly dynamic and the Golgi ribbon can undergo rapid changes in morphology^15,16^. Of relevance to the discussion here on mTORC1, modulation of the Golgi architecture is associated with the regulation of a number of signalling pathways^17^. It is now clear that there is an intimate relationship between the molecular pathways that regulate the dynamics of the architecture and morphology of the Golgi and signalling (see reviews 17,18). Studies have revealed that the Golgi provides a platform for the regulation of a range of cellular processes, including cell polarisation^19^, directed migration, stress^20^, mitosis, metabolism^21^, pro-inflammatory responses^22^ and autophagy^23^. Morphological changes of the Golgi are associated with the regulation of these cell processes (see review 17) and some of these are linked to mTOR signalling.

## Location of mTORC1 components on the endoplasmic reticulum/Golgi

There are a number of reports identifying mTORC1, and other components associated with activation of mTORC1, at the Golgi. The mTOR polypeptide, the kinase subunit of the mTOR complexes, has been located at the Golgi^24–26^. Our study in particular^26^ used an extensive range of organelle markers and fixation conditions and provided strong evidence for a functional pool of mTOR at the Golgi and particularly enriched at the *tran*s-Golgi network (TGN). The specificity of staining was demonstrated by knockdown of mTOR. Phosphorylated mTOR, representing the active mTOR, was also detected on Golgi membranes, and the phosphorylation was inhibited by the selective mTORC1 inhibitor, rapamycin, indicating that active mTORC1 was present on Golgi membranes^26^.

Other components associated with the activation of mTORC1 have been located at the endoplasmic reticulum (ER) and Golgi. The GTPase Rheb is an essential and immediate upstream activator of mTORC1^27^. Rheb is recruited from the cytosol to membranes, mediated in part by farnesylation of the Rheb C-terminal CaaX motif^28,29^. Inhibition of active GTP-bound Rheb results in suppression of mTORC1 activity^30^. Given the requirement of Rheb to activate mTORC1, the location of this small GTPase is also relevant. Rheb has been reported to be associated with a number of endomembranes, including the Golgi in different cell types^29,31,32^. It is currently unclear whether Rheb has specific membrane-targeting motifs or whether Rheb binds to membranes non-selectively^31,33^.

**Figure 1.**
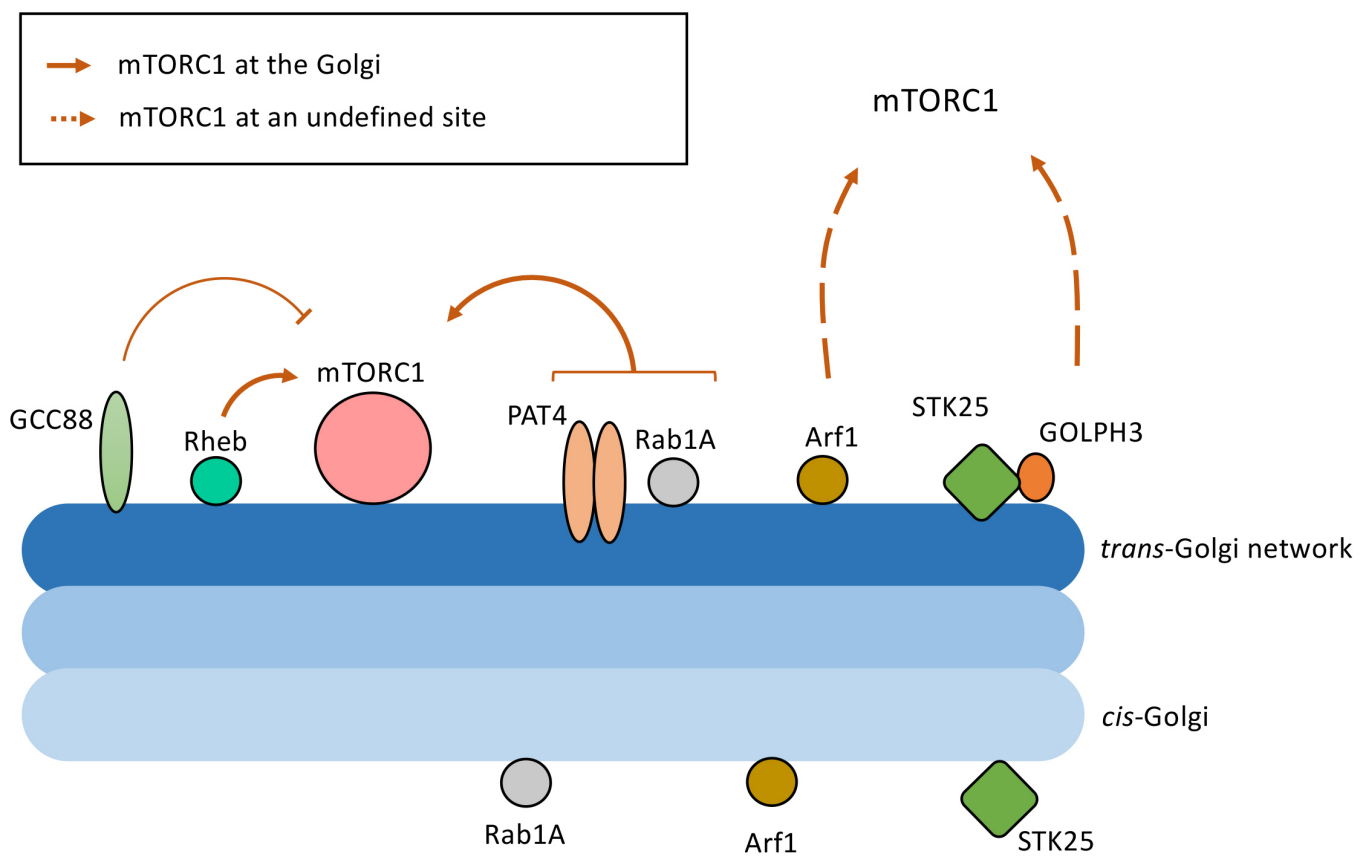
Golgi-localised components that mediate mechanistic (or mammalian) target of rapamycin complex 1 (mTORC1) signalling. Shown are components of the TORC1 pathway located at the Golgi. All of the components are located at the *tran*s-Golgi network. Some components are also located at the *cis-*Golgi, as indicated. The components that have been reported to regulate mTORC1 activity at the Golgi are indicated by solid lines, whereas other components that activate mTORC1 at a location yet to be defined are indicated by broken lines. The mechanisms by which the Golgi components regulate mTORC1 remain poorly understood.

The small GTPase, Rab1A, a component of the ER-to-Golgi trafficking machinery, has also been identified as a regulator of mTORC1^24^. Rab1A overexpression promotes mTORC1 signalling and cell growth^24^. Using transfections systems and knockdowns, Rab1A was shown to promote Rheb–mTORC1 interactions at the Golgi apparatus in response to amino acid stimulation^24^. Notably, transporters at the Golgi, in particular the Golgi glutamine transporter PAT4, have also been shown to contribute to mTORC1 activation^34^. The small GTPase, Arf1, has also been implicated in the regulation of mTORC1 signalling by a Rag-independent mechanism^35,36^ and, although the study did not provide direct evidence of mTORC1 associated with the Golgi, Arf1 is a well-characterised Golgi-localised small GTPase, located at both the TGN and *cis*-Golgi/ER-Golgi intermediate compartment^37^. Hence, mTOR and components of the activation pathway are present at the Golgi or Golgi/ER interface (or both), as illustrated in Figure 1.

## Evidence that mTORC1 signalling is mediated by Golgi-localised components

Identification of the components of the mTORC1 pathway at the Golgi demonstrates a contribution by this organelle to the mTOR pathway. However, localisation *per se* does not demonstrate that activation of mTORC1 occurs on Golgi membranes. In light of the localisation studies alone, it is formally possible that the Golgi represents a reservoir of mTORC1 pathway components and that activation occurs at other sites. However, a growing body of work demonstrates the ability of the Golgi apparatus to directly regulate mTOR activity, independently of lysosomes.

First, the activation of mTORC1 by Rab1A was demonstrated to be independent of lysosomes. Knockdown of Rab1A abrogates the Rheb–mTORC1 interaction at the Golgi but does not interfere with the interaction of mTORC1 with Rag on lysosomes^24^. Moreover, mTORC1 activation at the Golgi via Rab1A could rescue defects in mTORC1 activation at the lysosomes and vice versa, which argues that the Rab1A and Rag small GTPases are independently regulating mTORC1 at different locations^24^.

Second, our work found that the organisation of the Golgi as a ribbon structure is essential for regulating the mTORC1 pathway and autophagy. Conversion of the Golgi ribbon into Golgi mini-stacks, mediated by the increased expression of the Golgi membrane tether GCC88, which links Golgi membranes to the actin cytoskeleton at the TGN, resulted in an increase in LC3-positive autophagosomes^26^. The level of phosphorylated ribosomal S6 in HeLa cells which lack a Golgi ribbon is reduced compared with wild-type HeLa cells^26^. In addition, there was a significant reduction of both mTOR and p-mTOR^26^ on the scattered Golgi mini-stacks of HeLa cells lacking a Golgi ribbon compared with wild-type HeLa cells. Using balifomycin A1, which interferes with mTOR recruitment to lysosomes, we also demonstrated that mTOR could be recruited to Golgi membranes in the absence of a lysosomal mTOR pool, indicating that the Golgi and lysosomes have independent mTOR pools.

Third, *in situ* proximity ligation analysis yielded a positive signal between the glutamine transporter PAT4 and both mTOR and Rab1A, revealing that mTORC1 is located on the Golgi^34^. In addition, glutamine has been reported to stimulate mTORC1 by a Rag-independent mechanism^35^, indicating additional pathways of activation. Glutamine and serine activation of mTORC1 by the SLC36 transporter PAT4 shows preference for the phosphorylation of the downstream target 4E-BP1 compared with ribosomal S6, indicating that the Golgi activation pathway may be selective^34^. These findings suggest that mTORC1 may regulate different downstream pathways at different intracellular locations.

Fourth, mTOR signalling is modulated by Golgi phosphoprotein 3 (GOLPH3), another Golgi membrane tether that links the Golgi membranes to actin cytoskeleton and that has a role in the maintenance of the Golgi structure^38,39^. Increased levels of GOLPH3 result in enhanced mTORC1 activity and reduced autophagy^40,41^. Alterations in the level and phosphorylation of GOLPH3 result in changes in Golgi morphology, from the dispersal of Golgi fragments and the loss of the Golgi ribbon mediated by elevated GOLPH3 levels to an enhanced compaction of the Golgi morphology where levels of GOLPH3 are reduced^38,40,41^. The mechanism whereby GOLPH3 activates mTORC1, by a rapamycin-sensitive manner, is unclear, and a more extensive analysis of the GOLPH3 binding partners is required to resolve this issue.

Fifth, other Golgi proteins have been reported to modulate mTOR activity. Clem16A is a Golgi protein, which was recently reported to negatively regulate autophagy by activating mTOR^42^, and GOLPH2 (gp73, GOLM1) has been reported to promote mTOR signalling via the PI3K/AKT pathway^43^. In addition, the Ser/Thr protein kinase, STK25, a Golgi-localised kinase that regulates Golgi morphology and interacts with GOLPH3, downregulates mTORC1 activity and suppresses cell proliferation^44^. Members of the SLC38 amino acid transporters, namely SNAT2 and SNAT10, are Golgi-localised under certain conditions and have been reported to influence mTORC1 activity^45–47^. However, the intracellular location of mTORC1 activation via these transporters is not clear.

Collectively, the above indicates that there may be multiple pathways by which the Golgi can regulate mTOR signalling, independent of lysosomes. There is now substantial evidence for a functional pool of mTOR at the Golgi and the architecture of the Golgi influences mTOR activation.

## Golgi–organelle membrane contact sites and mTORC1

As discussed above, different morphological states of the Golgi architecture influence mTOR activation. The membrane tethers of the Golgi which influence Golgi architecture are known to interact with a number of components, and we have previously suggested that these membrane tethers may be akin to scaffold molecules to recruit a diverse set of components, including signalling machinery^48^. Indeed, many of the Golgi membrane tethers bind to small G proteins, which are essential regulators of signalling pathways. Another important consideration which could influence not only mTORC1 signalling but also other signalling pathways is the integrity of membrane contact sites between the Golgi and other intracellular organelles. Golgi membranes, and in particular the TGN, are known to make contacts with the ER and late endosomes/lysosomes^49–51^. Membrane contact sites between organelles mediate a number of processes, including the transfer of lipids, which in turn regulates the recruitment of cytolosic proteins to membranes. Some reports indicate that the majority of the small GTPase, Rheb, is localised to the Golgi^32,52^ and that membrane contact sites between the Golgi and lysosomes may be required for lysosomal activation of mTORC1^32,51^. Therefore, the Golgi should also be considered in the context of its membrane contacts with other organelles. An important issue to be investigated is whether the changes in the dynamics of the Golgi morphology modulate the membrane–membrane contacts sites between the Golgi and the ER or lysosomes and, if so, how the dynamics of these membrane contacts affect the recruitment and activation of mTORC1. At this stage, it remains unclear whether activation of mTORC1 is a co-ordinated process between the Golgi and lysosomes and whether changes in Golgi morphology have an indirect effect on lysosomal mTORC1 activation.

## Golgi, oncogenes, mTOR and cancer

A number of the Golgi components that influence mTORC1 signalling promote cell growth and tumourigenesis. Rab1A and GOLPH3 have been demonstrated to be oncogenes and are upregulated in a number of human cancers, including colorectal, prostate and gastric cancers^24,40,41,53–56^. Rab1A overexpression promotes mTORC1 signalling and also oncogenic growth in *in vitro* and *in vivo* model systems^24^. Overexpression of GOLPH3 has been shown to enhance cell survival following DNA damage^40^. The DNA damage response triggered by clinical therapies, which protects tumour cells from apoptosis, is regulated by GOLPH3. The nuclear kinase, DNA-Pk, which is activated following therapies, phosphorylates GOLPH3 and promotes loss of the compact Golgi ribbon, enhanced Akt1-mTOR signalling and cell survival^40,57^. In contrast, depletion of GOLPH3 maintains a compact Golgi following treatment with DNA damaging agents, increases apoptosis and reduces cell survival^40^. Recently, small non-coding RNAs, microRNAs (miRNAs), which have a protective role in preventing Golgi fragmentation mediated by GOLPH3 have been identified. These miRNAs, namely 3135b and 3150b-3p, have been shown to regulate the expression of GOLPH3^58,59^. For example, transfection of miRNA-3135b in HCT-15 cancer cells sensitised the cells to DNA damage by downregulating the expression of GOLPH3, resulting in reduced levels of mTORC1 signalling^58^. Hence, miRNA-3135b is a tumour suppressor regulating mTORC1 signalling via GOLPH3-mediated changes in Golgi architecture. Notably, miRNA-3135b and miRNA-3150b-3p are downregulated in colorectal cancer cell lines^58,59^. These findings are important as they indicate that the regulation of mTOR signalling via Golgi pathways is fundamental for normal cellular homeostasis and that aberrant Golgi-mediated signalling promotes tumourigenesis and also escape of cancer cells from treatment by chemotherapy.

## Conclusions

The evidence presented here strongly indicates a direct role for the Golgi apparatus in regulation of mTORC1 signalling in a diverse range of mammalian cells, including cultured epithelial and neuronal cells, *in vitro* tumour cells and *in vivo* colorectal cancers. Perturbation of these Golgi-mediated mTORC1 pathways has been demonstrated to be relevant for tumourigenesis. The dynamic nature of the structure of the Golgi and the ability to recruit peripheral membrane proteins to specific subdomains of the Golgi membrane^60^ make the Golgi an ideal template to rapidly modulate the strength of mTOR signalling. In addition, the Golgi has a direct role in regulating the biogenesis of autophagosomes^61^ and, given the negative regulation of autophagy by mTORC1 signalling, the Golgi represents a site for the co-ordination of both pathways. Currently, the information on mTORC1 activation at the Golgi has been obtained from biochemical, cell biological and microscopic techniques. Biophysical approaches now need to be applied to visualise the activation of mTORC1 in real time at specific intracellular locations and in a range of different primary cells. This approach will also provide the ability to determine which inputs/sensors activate the Golgi mTORC1 signalling pathway. Do both intrinsic and extrinsic sensors, or only intrinsic sensors, activate Golgi mTORC1, and which downstream pathways are activated by Golgi mTORC1? In addition, further consideration should be given to whether mTOR is activated at intracellular locations in addition to lysosomes and the Golgi.
